# Risk factors for H5 avian influenza virus prevalence on urban live bird markets in Jakarta, Indonesia—Evaluation of long-term environmental surveillance data

**DOI:** 10.1371/journal.pone.0216984

**Published:** 2019-05-24

**Authors:** Joerg Henning, Uta Walburga Hesterberg, Farida Zenal, Luuk Schoonman, Eric Brum, James McGrane

**Affiliations:** 1 School of Veterinary Science, University of Queensland, Gatton, Queensland, Australia; 2 Emergency Centre for Transboundary Animal Diseases (ECTAD), Food and Agriculture Organization of the United Nations (FAO), Jakarta, Java, Indonesia; 3 Emergency Centre for Transboundary Animal Diseases (ECTAD), Food and Agriculture Organization of the United Nations (FAO), Dhaka, Bangladesh; The University of Hong Kong, CHINA

## Abstract

In the re-emergence of Highly Pathogenic Avian Influenza (HPAI), live bird markets have been identified to play a critical role. In this repeated cross-sectional study, we combined surveillance data collected monthly on Jakarta’s live bird markets over a five-year period, with risk factors related to the structure and management of live bird markets, the trading and slaughtering of birds at these markets, and environmental and demographic conditions in the areas where the markets were located. Over the study period 36.7% (95% CI: 35.1, 38.3) of samples (N = 1315) tested HPAI H5 virus positive. Using General Estimation Equation approaches to account for repeated observations over time, we explored the association between HPAI H5 virus prevalence and potential risk factors. Markets where only live birds and carcasses were sold, but no slaughtering was conducted at or at the vicinity of the markets, had a significantly reduced chance of being positive for H5 virus (OR = 0.2, 95% CI 0.1–0.5). Also, markets, that used display tables for poultry carcasses made from wood, had reduced odds of being H5 virus positive (OR = 0.7, 95% CI 0.5–1.0), while having at least one duck sample included in the pool of samples collected at the market increased the chance of being H5 virus positive (OR = 5.7, 95% CI 3.6–9.2). Markets where parent stock was traded, were more at risk of being H5 virus positive compared to markets where broilers were traded. Finally, the human population density in the district, the average distance between markets and origins of poultry sold at markets and the total rainfall per month were all positively associated with higher H5 virus prevalence. In summary, our results highlight that a combination of factors related to trading and marketing processes and environmental pressures need to be considered to reduce H5 virus infection risk for customers at urban live bird markets. In particular, the relocation of slaughter areas to well-managed separate locations should be considered.

## Introduction

**Fig 1:**
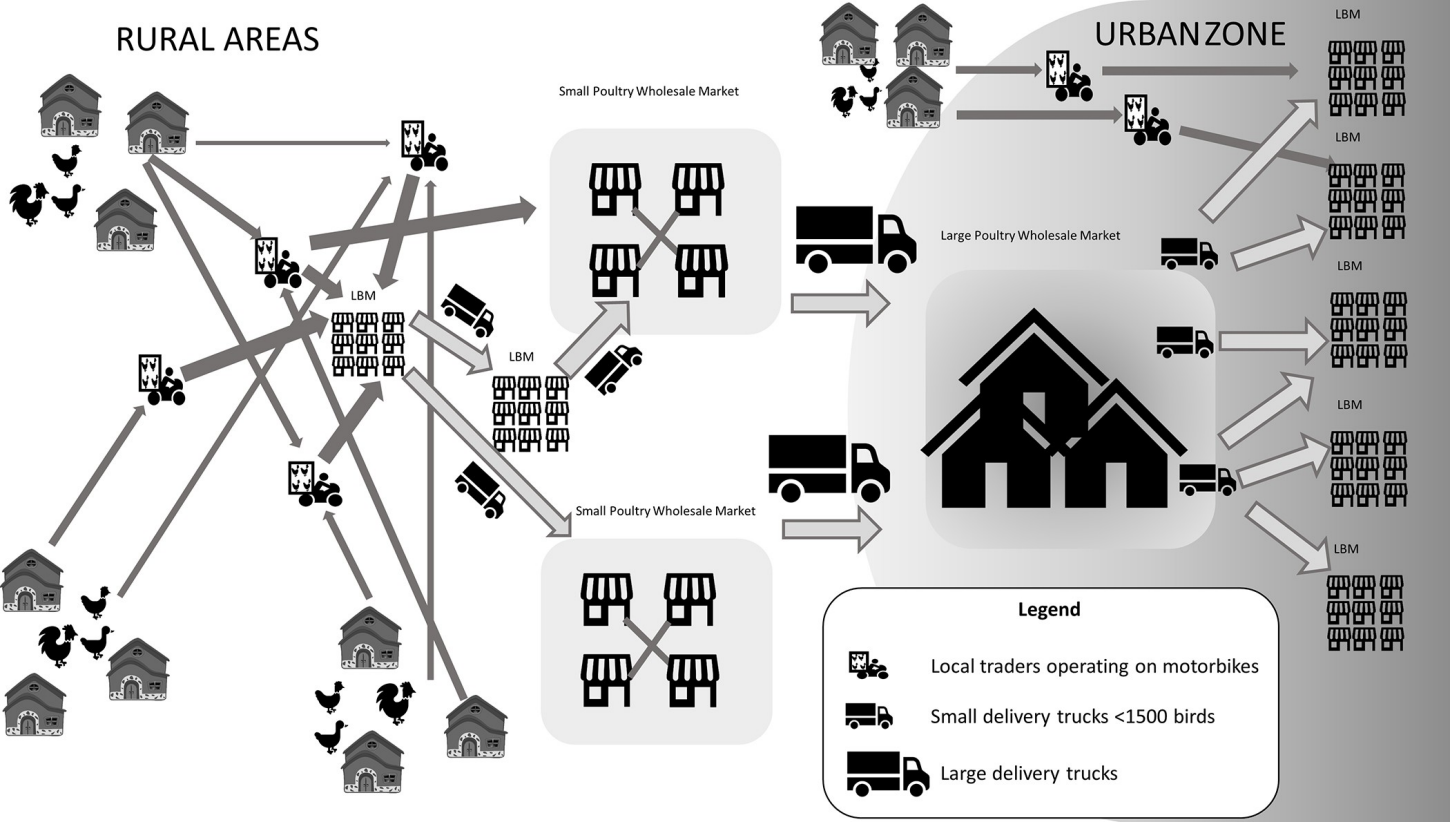
Value chain for backyard poultry traded from producers to live bird markets in Indonesia.

**Fig 2:**
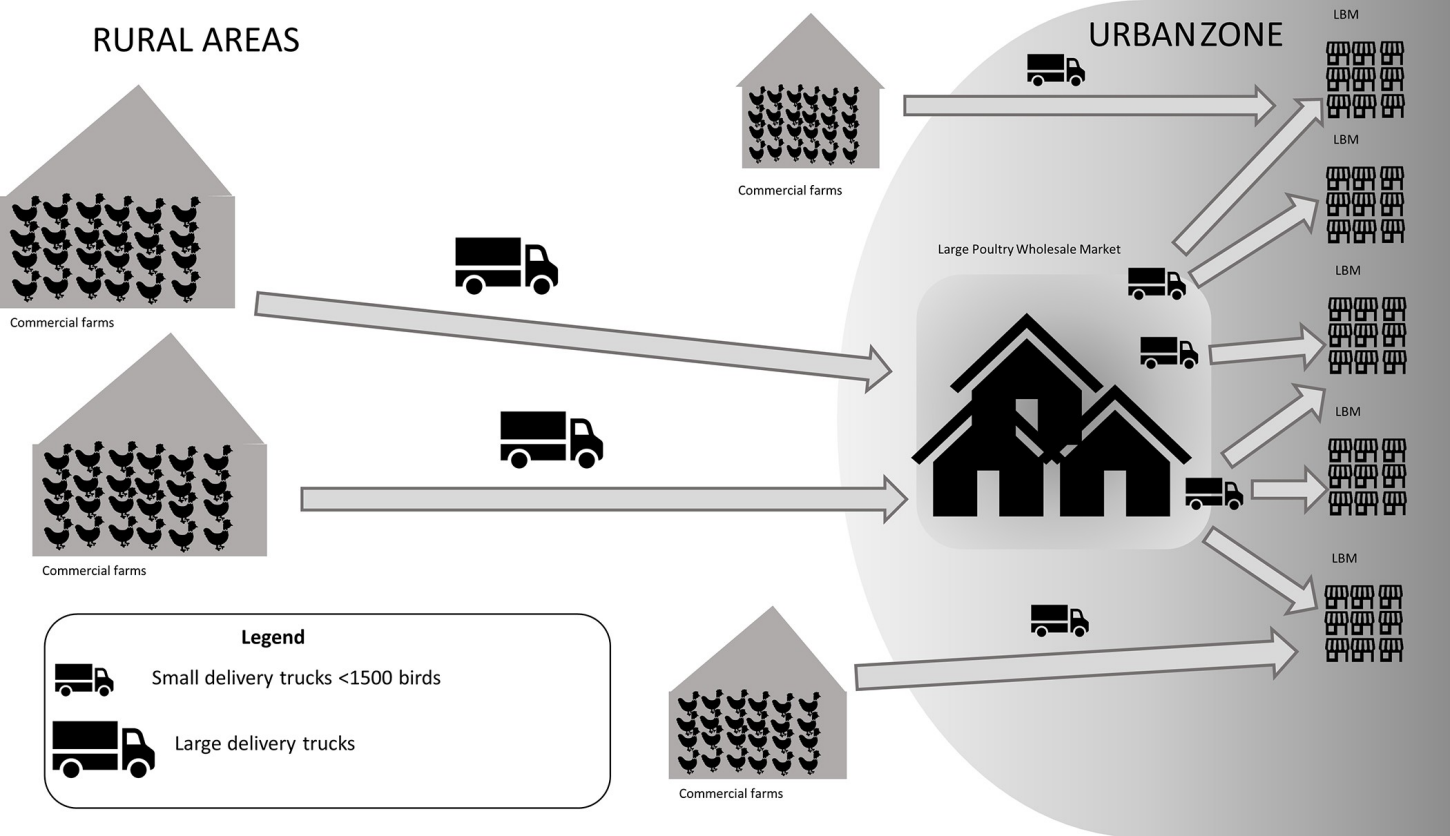
Value chain for commercial broilers traded from producers to live bird markets in Indonesia.

Since the first wave of highly pathogenic H5N1 (HPAI H5N1) occurred in 2003/04 in South East Asia, the virus has managed to maintain itself in several countries, resulting in an endemic state and regular disease outbreaks. Especially in the early years of the incursion of HPAI H5N1, many human cases were reported, arousing fears of an emerging human pandemic. By far, Indonesia reported the highest number of human cases in the region. Interestingly, many human cases were not reported from areas of high poultry density, but rather from urban areas in which hardly any poultry is produced. Investigations revealed that most human cases of not only HPAI H5N1, but also of the more recently discovered strains H7N9 and H10N8, have been linked to visits of live bird markets (LBM) [1–5]. LBMs provide live poultry that is frequently slaughtered at the markets and sold to the public or small restaurants. About 70–80% of poultry meat in Indonesia is distributed through such traditional LBMs with slaughter facilities [6]. Results from initial surveillance activities implemented in Indonesia revealed a much higher prevalence of HPAI H5N1 at these LBM compared to poultry producing areas, indicating that HPAI virus must spread extensively during the trading process. However, there are important differences in the value chain of backyard poultry (e.g. Kampung or indigenous chickens) and commercial poultry (e.g. broilers and layers) sold in urban LBMs in Indonesia (Figs 1 and 2).

Backyard poultry is usually purchased by ‘middlemen’ or small-volume poultry traders who visit several villages on motorbikes to trade with farmers or they purchase birds from small village markets. They then sell these birds at LBMs in rural areas, from where birds are transported to smaller poultry wholesale markets. There, birds are re-assorted and mixed according to size, before being transported to larger wholesale poultry markets and further distributed to LBMs in urban centres (Fig 1). Thus, the time a backyard chicken might spend in transport can exceed 30 hours (Food and Agriculture Organisation, unpublished data). For broilers, the trading chain is less complex and shorter (Fig 2). Most broilers are purchased by so-called brokers and subsequently transported by trucks to urban poultry wholesale markets for re-assortment and subsequent sale at urban LBMs. A smaller proportion of owners of broiler flocks sell their birds directly to LBMs; these are mainly flock owners with flock sizes of less than 5,000 birds. It has been estimated that 70% of the total broiler meat in Indonesia is produced through contract farms, 20% by independent farms and 10% by large integrated farms [7].

Therefore, the high HPAI virus prevalence observed at LBM is probably related to the duration poultry remains in the trading chain before being sold at LBM and is influenced by the number and frequency of contacts of susceptible birds with infected poultry or with HPAI virus contaminated surfaces. Since it is difficult to acquire data explaining these associations, conclusion on the impact of trading on HPAI virus spread are mainly based on infectious disease modelling [8, 9] supported by empirical evidence describing varying HPAI prevalence on farms, at wholesale and at retail markets [10, 11]. The connectivity of LBMs has been described as a factor impacting on LBM H5 virus prevalence [5], probably representing the movements of poultry traders between LBMs. Particularly in areas with high LBM density, movements are likely to be shorter and consequently the HPAI virus might possess a greater chance of staying viable during these movements. Additionally, virus survival in the market environment, such as in poultry cages, drinking water and water puddles has to be considered [10]. Also since birds frequently stay overnight at markets, these birds might become a “reservoir” for HPAI virus spread [9, 10, 12, 13]. Research studies on LBMs in Indonesia have identified slaughtering of poultry at the LBM as an factor that increased the risk of a LBM being tested positive for HPAI H5N1 [13]. Factors that reduced the risk of HPAI H5N1 at LBMs included clear zoning with separation of sale and slaughter and daily waste disposal [13, 14]. Studies in China supported that effective waste disposal and regular disinfection helped to reduce the risk of HPAI infection [15, 16].

However, most of these studies focussed only on one group of risk factors, either market characteristics or environmental and infrastructural conditions that might be associated with HPAI virus prevalence while using cross-sectional diagnostic data collected only at a single time point.

In our study, we combined regular surveillance results collected repeatedly from LBMs in the Greater Jakarta region, Indonesia, over a period of five years with information on market characteristics, trading parameters, market density, environmental conditions and sampling characteristics to provide holistic insights into the HPAI H5 infection dynamics at LBMs.

## Materials and methods

### Environmental LBM surveillance, sample collection and HPAI H5 virus testing

The data collection was conducted in the 13 districts of the Greater Jakarta region, which included the following: Bekasi, Bogor, Jakarta Barat, Jakarta Pusat, Jakarta Selatan, Jakarta Timur, Jakarta Utara, Kota Bekasi, Koa Bogor, Kota Depok, Kota Tangerang, Tangerang and Tangerang Selatan. The Government of Indonesia, in collaboration with the Food and Agriculture Organisation (FAO), has been conducting environmental LBM surveillance since 2009. It involves the collection of environmental samples from individual vendors. A vendor represents a single retailer who sells live birds or poultry carcasses to customers and who may or may not slaughter birds on site. The number of vendors in a market can vary greatly, between one and more than 50 (Food and Agriculture Organisation, unpublished data). Initially, surveillance of LBMs in Jakarta included all of the 260 identified LBMs, but to reduce the costs associated with these activities, surveillance was reduced to 86 LBMs in 2012. A risk-based approach was used to select LBMs that should undergo further surveillance: LBMs were ranked according to their completeness of surveillance data and frequency of HPAI H5 positive results, and by ensuring that a good geographic coverage of all districts in the Greater Jakarta region is maintained (with the number of markets under surveillance in each district being proportional to the total number of live bird markets being present in the respective district).

LBM’s were visited approximately monthly by a government official and a pooled environmental sample was collected from six vendors, with one environmental swab obtained per vendor. Monthly collection of pooled samples commenced in March 2009, but in May 2012, sample collection was changed from a monthly to a quarterly protocol, and returned to monthly sampling in February 2013.

The sample sites for the pooled samples were selected based on research by Indriani et al. 2010, who tested a multitude of environmental sampling sites at markets and found six locations to be the most sensitive for the detection of H5N1 [13]. The environmental sampling conducted with swabs included the following locations: 1) the cutting board, 2) the de-feathering drum, 3) the waste bins, 4) the cutting knife, 5) the cleaning cloth and 6) the display surface. Government officials collecting the samples were instructed to change the vendors they select for sampling every month, without applying a specific pattern. If no slaughtering was conducted at the LBM, swabs were collected from the display areas of poultry carcasses, including processing and cutting boards, as well as knives used to process carcasses. Pooling of the six swabs in one tube containing virus transport medium was conducted at the LBM. Pooled samples were kept in ice boxes and transported to the DKI Jakarta Animal Health Laboratory (BKHI laboratory) on the same day. Samples were stored at -20° C for a maximum period of 60 days.

RNA extraction was carried out using Qiamp^R^ Viral RNA Mini Kit (Qiagen inc.). The RNA template was used for Influenza A virus detection (M gene) with the Influenza Virus Type A & Avian Influenza Virus TaqMan PCR assay [17]. A H5 subtype PCR was conducted if a sample tested M-gene positive. Until 2014, H5 testing was conducted using a conventional PCR and was later changed to Real Time PCR. All testing protocols followed the procedures recommended by the Australian Animal Health Reference Laboratory (AAHL) [17].

### Risk factor data collection

Risk factor information on LBM trading, poultry processing and selling practices was collected through questionnaire surveys conducted between 2008 and 2014 on LBMs in the Greater Jakarta region. Questionnaires used in the surveys were pilot-tested with at least five interviewees and modified if required. The government officials conducting the surveys were trained in interviewing techniques for each survey during one to two-days workshops. The following surveys were conducted:

1. A survey of poultry markets (wholesale and retail) was carried out in 2008 to describe the trading volume and practices (Food and Agriculture Organisation, unpublished data). The geographical locations of these poultry markets were used to calculate their density (number of markets/square km) for each of the districts in the Greater Jakarta region. The information was also used to calculate the average distance between the centroid of districts supplying the poultry and the district centroid of the market (representing the average distance for transportation of poultry to the LBM).
2. A survey of all the 86 LBM’s under surveillance was carried out in March 2013 by government officials, as part of the routine monthly surveillance activities. The survey was designed by FAO Indonesia and explored general trading information, data on volumes and species of poultry traded and data on slaughtering facilities (Food and Agriculture Organisation, unpublished data).
3. A follow-up survey was conducted in February 2014 by local government officers during the monthly surveillance activities. Data were collected on the market layouts, positioning of live bird holdings, slaughtering facilities and slaughtering and cleaning practices, as well as implementation of control and biosecurity practices. Subsequently, the physical structure of LBMs was categorized. Since LBMs differ significantly with regard to their layout of slaughtering and live bird storage facilities, the eight most common designs were captured in reference sketches in a participatory approach with government officials involved in the surveillance activities. Markets were then categorized as per the layout sketch that most resembled their structure.

A total of 22 risk factors potentially influencing HPAI H5 virus prevalence were compiled from the survey data. This included data on market characteristics (district of market location, market layout, most dominant poultry species traded and trading volume), data on the poultry management at the markets (material of poultry cages, methods used for stacking of poultry cages, material of display tables, type of surface area used for slaughter and if birds were staying overnight on the market), density of poultry retail and wholesale markets and average distance between market and location from where poultry was supplied to the market (kilometre). In addition, total monthly rainfall (mm) data for each month in the study period for Jakarta was sourced from “Weather Underground” (https://www.wunderground.com). Furthermore, data on the specific characteristics of each pooled sample were recorded: if samples were obtained from broilers only, if at least one sample of the pool was obtained from a Kampung chicken, if at least one sample of the pool was obtained from a duck, if samples were obtained from the slaughter or from the display area only. Thus a total of 29 risk factors were compiled.

### Statistical analysis

If a LBM pool tested H5 virus positive, the market was considered as H5 positive for this sampling occasion. LBM H5 virus prevalence was calculated by the dividing the number of positive market pools obtained per month by the total number of markets sampled in that month. Furthermore, the LBM H5 virus prevalence for the whole observation period was calculated. Binominal exact Clopper-Pearson confidence intervals were calculated for all prevalence values [18].

Since the same LBM’s were sampled multiple times over the study period, Generalized Estimating Equations (GEE) were used to account for this within-subject correlation. GEE models are a suitable solution for longitudinal panel data where multiple results are recorded for one subject and are therefore likely to be correlated, like the HPAI H5 virus status of sampled LBMs over time. The correlation matrix in GEE models represents the within-subject dependencies and parameter estimation is conducted using an iterative quasi-likelihood estimation. We used an exchangeable correlation structure in our model as it produced the best fit, although GEE models are very robust to correlation misspecification [19]. A weighted GEE was implemented, where the ‘N of observations/N of time periods for each market’ was the probability weight in the analysis [20, 21]. A binominal distribution with a logit link function was used to model the H5 virus status of LBMs. Standard errors were estimated using ‘robust’ Huber/white/sandwich estimators of variance. Continuous predictor variables were standardized by subtracting the mean from each value and then dividing it by the standard deviation, resulting in a mean of zero and a standard deviation of one for the standardized variables.

We started the analysis with a univariate analysis. Variables with p<0.1 in the univariate analysis were considered for the multivariable analysis. We also evaluated correlations between variables using the Pearson correlation coefficient for continuous variables and tetrachoric and polychoric correlations for dichotomous and categorical variables. If two predictors were highly correlated (>0.7), only one of the variables was used in the multivariable model. We used a backward selection processes to develop the final model with predictors significant at p<0.05. Generalised joint Wald tests were used to test the significance of each fitted categorical variable with more than two levels. All reported p-values are two sided. Stata 14.0 (Stata Statistical Software, Stata Corporation, College Station, TX, USA) was used for the statistical analyses. Categorized period prevalence values were plotted for each market location using ArcGIS 10.4 (Esri Inc).

## Results

### Live bird market HPAI H5 virus prevalence

A total of 4,213 pooled LBM samples were collected from 86 LBMs between March 2009 and July 2014. Although all the 86 LBMs should have been sampled every month, in practice not all were sampled monthly. Over the study period of 65 months, a single LBM was sampled on average 51 times ranging from 17 to 59 times. Complete risk factor information and diagnostic data was available for 79 LBMs, representing 3,579 pooled LBM samples, which were used for the analysis. LBMs were trading on average 4,739 birds per day ranging from 65 to 102,900. The average number of vendors per market was 17, ranging from 1 to 64.

**Fig 3:**
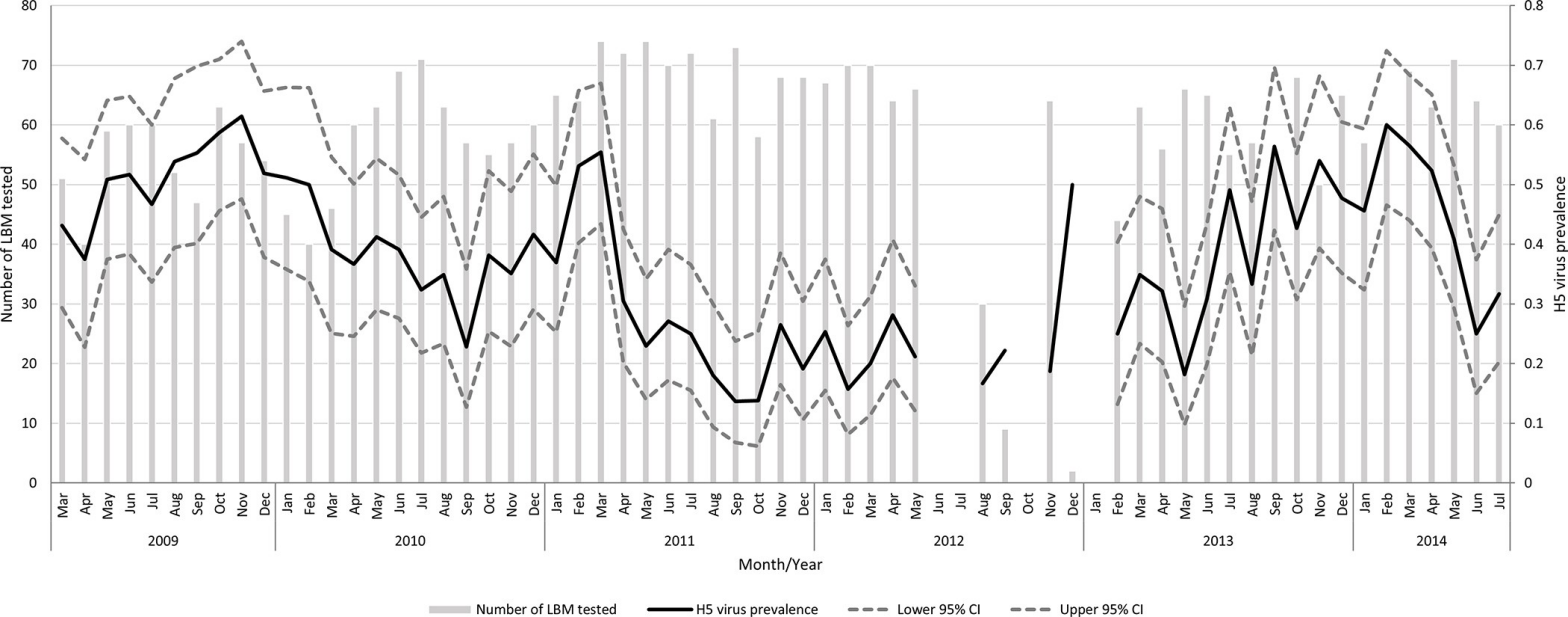
Number of live bird markets tested and HPAI H5 virus prevalence per month in the Greater Jakarta region, Indonesia, between March 2009 and July 2014.

Over the whole study period 36.7% (95% CI: 35.1, 38.3) (N = 1,315) LBM samples tested HPAI H5 virus positive. The number of LBMs tested and the monthly HPAI H5 virus prevalence (including 95% confidence intervals) are displayed in Fig 3. A strong seasonal pattern was observed with a higher proportion of LBMs testing H5 virus positive in the 2^nd^ half of the year, peaking between October-February in 2009, 2010, 2011 and also in 2013. The seasonal peak of the October-February period in 2011 was lower than in the previous years (by only about 20%). As LBM sampling was infrequently conducted in the 2^nd^ part of 2012, clear seasonal patterns could not be observed. During the high risk period of 2013, LBM H5 virus prevalence again reached the 2009 values of about 50%.

**Fig 4:**
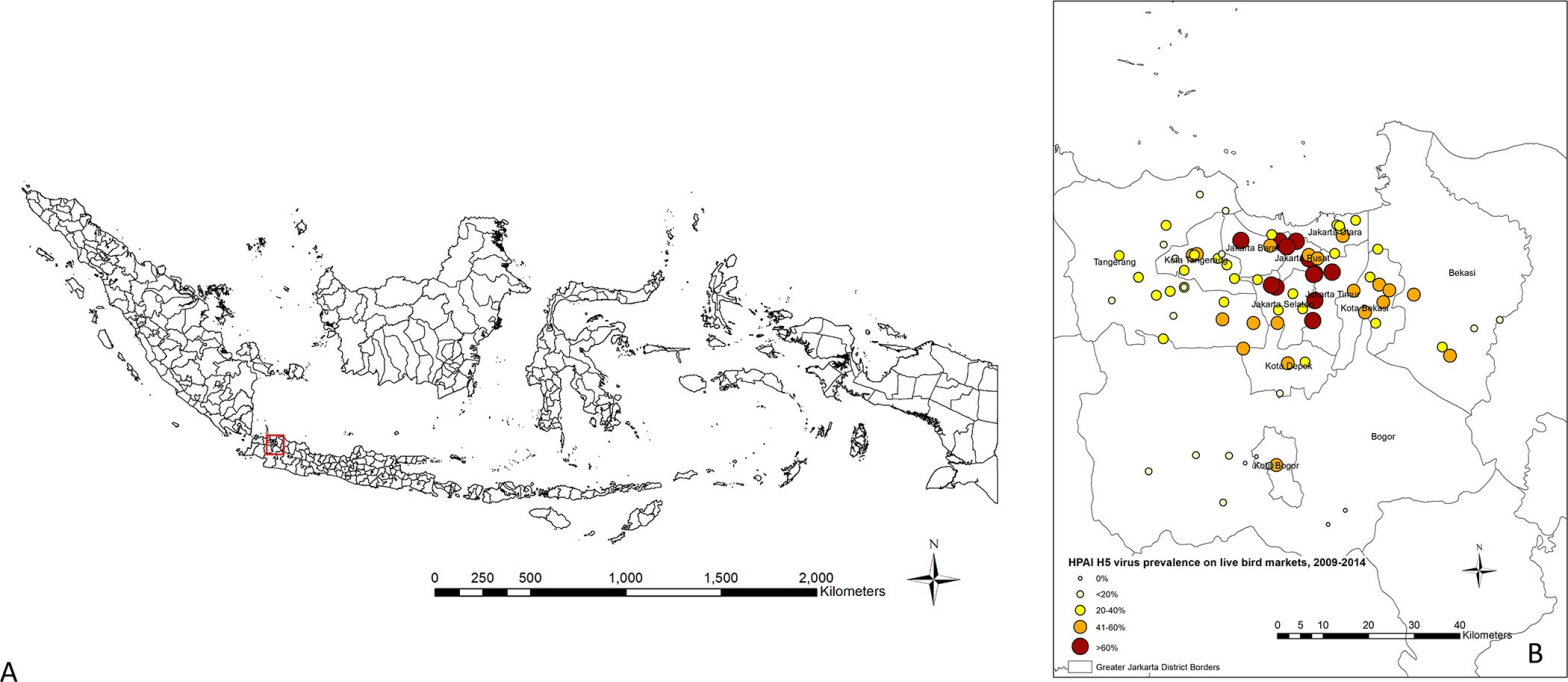
H5 virus period prevalence by live bird market location in Greater Jakarta, Indonesia. A: Map of Indonesia with the Greater Jakarta region highlighted in red square. B: Categorized HPAI H5 virus period prevalence by live bird market location in the Greater Jakarta region between March 2009 and July 2014.

There was a considerable variation between districts (S1 Table) with Jakarta Timur LBMs having the largest number of positive samples per district over the study period (78.6%, N = 191) and Bogor LBMs having the lowest (6.3%, N = 22). Fig 4 shows the categorized H5 virus period prevalence (2009–2014) by LBM location over the whole study period.

### Risk factors for live bird market HPAI H5 virus positivity

The proportion of HPAI H5 virus positive and negative samples, the odds ratios for being H5 virus positive (including 95% confidence intervals) and the p-values for all risk factors evaluated are displayed in S1 Table. Twenty-one variables were statistically significant at p<0.1 in the univariate analysis and were considered for the multivariable model.

After exploring bivariate correlations between potential risk factors, we excluded one of the two risk factors with a correlation of larger than 0.7. These included *Samples from slaughter area only* (correlated with *Slaughter at the market*) and *Market location* (correlated with *Density of poultry retail markets in the district* and *Average distance between market and origin of poultry sold at market)* and *Human population density*) and *Density of poultry wholesale markets in the district* (correlated with *Human population density*).

The final multivariable model included risk factors related to the market characteristics, the poultry management on the market, the sampling conducted at the market and some environmental factors (Table 1).

**Table 1:** Final multivariable model results for risk factors associated with HPAI H5 virus prevalence at live bird markets in the Greater Jakarta Region, Indonesia, between March 2009 and July 2014.

Risk factor	Level	N Observations (percent)		Univariate analysis			Multivariable analysis		
		H5 negative	H5 positive	OR	P-value	Wald test P-value	OR	P-value	Wald test P-value
**MARKET CHARACTERISTICS**	** **								
Market layout^1^	A	934 (64.3%)	518 (35.7%)	1		<0.001	1		<0.001
	B	537 (58.3%)	384 (35.7%)	1.3 (0.8, 2.3)	0.322		1.1 (0.7, 1.6)	0.786	
	C	169 (56.1%)	132 (43.9%)	1.3 (0.8, 2.0)	0.268		1.5 (1.0, 2.2)	0.060	
	D	33 (34.4%)	63 (65.6%)	3.3 (2.3, 4.9)	<0.001		1.4 (0.8, 2.3)	0.229	
	E	58 (57.4%)	43 (42.6%)	1.3 (0.7, 2.4)	0.358		1.1 (0.5, 2.2)	0.829	
	F	120 (79.5%)	31 (20.5%)	0.3 (0.1, 0.9)	0.03		0.4 (0.2, 1.1)	0.071	
	SALE only 1	159 (56.0%)	125 (44.0%)	1.4 (0.7, 2.8)	0.377		1.3 (0.7, 2.1)	0.417	
	SALE only 2	254 (93.0%)	19 (7.0%)	0.1 (0.0, 0.3)	<0.001		0.2 (0.1, 0.5)	0.002	
Most dominant poultry species on the market	Broilers	1,592 (66.4%)	807 (33.6%)	1		<0.001	1		<0.001
	Layers	92 (66.2%)	47 (33.8%)	1.0 (0.7, 1.5)	0.849		0.7 (0.5, 1.0)	0.054	
	Kampung Chickens	435 (56.3%)	337 (43.7%)	1.5 (0.8, 2.9)	0.243		1.3 (0.8, 2.2)	0.355	
	Ducks	120 (54.5%)	100 (45.5%)	1.6 (0.8, 3.0)	0.149		0.9 (0.5, 1.9)	0.868	
	Parent stock	25 (51.0%)	24 (49.0%)	2.0 (1.5, 2.7)	<0.001		5.7 (3.6, 9.2)	<0.001	
**POULTRY MANAGEMENT ON MARKET**								
Display tables made from wood	No	1,032 (58.3%)	739 (41.7%)	1					
	Yes	1,232 (68.1%)	576 (31.9%)	0.6 (9.3, 0.9)	0.018		0.7 (0.5, 1.0)	0.047	
**SAMPLING CHARACTERISTICS**								
Samples obtained from at least one duck	No	2,083 (65.8%)	1,083 (34.2%)	1					
	Yes	181 (43.8%)	232 (56.2%)	1.5 (1.1, 2.1)	0.019		1.6 (1.1, 2.3)	0.009	
		**Mean (SD)**	**Mean (SD)**						
		**H5 negative**	**H5 positive**						
**ENVIRONMENTAL FACTORS**									
Human population density in the district (N people/square kilometre)	9,018.3 (5,023.0)	11,958.9 (4,379.6)	2.2 (1.8, 2.7)^2^	<0.001		1.6 (1.3, 1.9)^2^	<0.001		
Average distance between market and origin of poultry sold at the market (kilometre)	16.8 (35.7)	35.9 (47.8)	1.6 (1.2, 2.0)^2^	<0.001		1.3 (1.1, 1.6)^2^	0.011		
Total rainfall per month (mm)	94.2 (87.1)	113.3 (94.4)	1.2 (1.2, 1.3)^2^	<0.001		1.3 (1.2, 1.4)^2^	<0.001		

^1^ Live Bird Market layouts: A = Slaughter, sale of live birds and carcass sales in same area, B = Slaughter and live bird sales conducted outside and carcass sales inside, C = Slaughter, sale of live birds and carcass sales in same areas, but separated by individual partitions, D = Slaughter and live bird sales in same areas inside, and separated from carcass sales by screens, E = Slaughter, live bird and carcass sales inside in separate areas, but no protective screens, F = Live birds sale outside and slaughter and carcass sales inside and in separate areas, Sale 1 only = No slaughtering at the market, but slaughter facility in vicinity of the market, Sale 2 only = No slaughtering at the market, and slaughter facility far away from the market

^2^ Odds ratios and confidence intervals are displayed for standardized values

Markets where only carcasses were sold, but no slaughtering was conducted at or near these markets (market layout: SALE 2 only), had a significantly reduced chance of being positive for H5 virus (OR = 0.2 (95% CI 0.1–0.5), p = 0.002). Also, markets where live bird sales were conducted outside the LBM, and slaughter and carcass sales were conducted in separated areas inside the LBM (market layout: F) had marginally lower odds of being infected with HPAI H5 virus (OR = 0.4 (95% CI 0.2–1.1), p = 0.071). Interestingly, markets where parent stock was traded or slaughtered (6.3%, N = 5), were more at risk of being H5 virus positive compared to markets where broilers were traded (OR = 5.7 (95% CI 3.6–9.2), p<0.001). Markets, that used display tables for poultry carcasses made from wood, had reduced odds of being H5 virus positive (OR = 0.7 (95% CI 0.5–1.0), p = 0.047), while having at least one duck sample included in the pool of samples collected at a market increased the chance of the sample being H5 virus positive (OR = 5.7 (95%CI 3.6–9.2), p<0.001). Finally, the human population density in the district, the average distance between market and the origin of the poultry sold at market and total rainfall per month were all positively associated with higher H5 virus prevalence.

## Discussion

Our study aimed to identify risk factors for HPAI H5 virus prevalence, by evaluating long-term surveillance data collected at urban live bird markets in Indonesia. Our results highlight the role of the poultry trading chain and its impact on the infections status of markets.

There is a strong relationship between human population density and the risk of markets being virus positive, also highlighted by the fact that district Jakarta Timur, with the highest population density in the Greater Jakarta region, had also the highest H5 virus prevalence over the study period. Human population density has been previously described as a risk factor when identifying HPAI in backyard village chickens through participatory disease surveillance in Indonesia [22], but detailed trade information such as wholesale market density was not available in the particular study. Another study found that when comparing correlated road density and human density with regard to their risk, road density provided the better indicator, leading to the conclusion that the risk is more likely to be attributable to trading activities [23].

In our study, we identified a strong correlation between human density and wholesale market density and therefore only included human population density in our multivariable model. However, the density of wholesale markets is likely to be an indicator for density of settlements and people living in an area as well as an indicator for connectivity between traders. The closer the wholesale markets are to each other, the shorter the transport time for poultry being traded between markets. It is also likely that the same traders will visit multiple wholesale markets to sell birds. Due to the large number of birds being traded at wholesale markets, each market will ‘provide’ a substantial source of new susceptible birds that can be infected by HPAI H5 virus. This association was recently highlighted by Gilbert et al. 2014, who found that market density was the most important predictor for market H7N9 prevalence [2]. Interestingly, we did not find a significant association between retail market density and the market infection status. Retail markets mainly provide live poultry or carcasses to customers and trading of poultry between traders at these markets is less common.

We also identified that the distance between the area where poultry originated and the LBM increased the chance of a LBM being infected. This observation supports the importance of disease spread during trading, heightened through many players being involved in the trade, as is the case with backyard chickens. The longer the distances and the more frequent the re-assortment of poultry, the more time the virus has to find new susceptible hosts.

Higher monthly rainfall also contributed to an increased chance of markets testing H5 virus positive. In wet conditions, the virus can survive for several days and this has been previously demonstrated to be of importance for virus persistence in LBMs [24]. This supports the observation of a higher H5 virus prevalence in the rainy season, which lasts between October and April. Often markets are only partly covered by solid roofs and their floors are not always made of solid material such as concrete. In rainy conditions, the market floors are likely to be wet and possibly muddy, increasing the virus survival rate, and enhancing the effect of virus accumulation in the market.

Overall, market layouts that did not have slaughtering conducted in the direct vicinity of the market had lower odds of being H5 virus positive (SALE 2 type of markets). During the slaughter process, body fluids are released and contaminate surface areas, floors and equipment providing a source for cross-contamination of H5 virus. Confining the slaughter to a separate location at the LBM, or even better far away from the LBM, where neither live poultry or displayed carcasses are present, is likely to reduce the risk of H5 spread and the infection risk for consumers.

Interestingly, there was a lower chance of samples being positive when display areas were made of wood. Although a previous study identified wooden tables as a risk factor for H5 virus market positivity [13], it did not distinguish between slaughter and display tables. Slaughtered poultry carcasses are usually displayed either on the floor or on tables made from wood, ceramic or stainless steel. The latter two provide smoother surfaces, that are probably easier to clean (an also had lower risk for H5 virus positivity in our study, but not significant at p<0.1). However, recent research has highlighted that the porosity of wood actually has a microbiological advantage [25]. Wood pores generate surface cavities that can trap bacteria in a state unfavourable for their survival, so bacterial growth is extremely limited. Moreover, the natural biofilms which form on wooden surfaces have been proven to be safe and able to inhibit pathogenic bacteria growth, although the underlying biological mechanisms need to be further explored [26].

Finally, our results demonstrated that if pooled samples collected contained at least one duck sample, then the chance of a market being H5 virus positive was also increased. The role of ducks with unapparent clinical signs of HPAI infection in the maintenance of HPAI H5 virus infection has being highlighted previously [27, 28] and a transmission of H5 virus from infected ducks to chickens or other poultry kept at LBM is likely. Although duck samples represented only 11.6% (N = 413) of the total number of samples collected in this study, a significant proportion of duck samples was positive. Interestingly, markets where parent stock was the most dominant poultry species (only 1.4% of markets), also has a higher chance of being H5 virus positive, probably because these older birds had a higher chance of becoming infected throughout their lifetime.

Our study used long term surveillance data in combination with data collected during cross-sectional studies. This implies that some risk factor variables were not regularly evaluated. It is possible that some biosecurity practices might have varied throughout the study period, but most risk factors we evaluated would have stayed the same (market layout, most dominant livestock species traded, materials of display tables etc.). However, other risk factors such as rainfall and type of samples were actually measured repeatedly throughout the study period (and proved to have a significant association with LBM H5 virus prevalence). We did not have suitable data available on the dedicated cleaning and disinfection programmes conducted at these markets. Cleaning and disinfection may reduce the virus survival in the market somewhat, but is unlikely to counter the effect of constant virus influx. Similar observations were made on LBMs in Bangladesh and New York respectively [14, 29]. Indiriani et al (2010) observed that frequent solid waste removal had a protective effect on H5 market status, but did not find other evidence of the impact of cleaning and disinfection [13]. All LBMs in our study operated seven days a week, so the impact of closing days could not be evaluated. This has been found to reduce the risk of market infection in other studies [11].

While our study design was not that of a conventional case-control study, it showed that surveillance conducted over a long time period can provide valuable insights into the risks associated with HPAI H5 infection at LBMs. Furthermore, our analysis included 79 out of the total of 260 LBMs in the Greater Jakarta area, which is approximately a third of all LBMs. This large geographical coverage strengthens the value of our findings, especially considering that markets had been tested on an average 51 times during the study period.

Overall, our diagnostic results mirror findings from other studies on LBMs in Asia and all significant risk factors identified are epidemiologically plausible. We therefore believe that our results can be extrapolated to countries with similar poultry trading systems.

In summary, our results indicate the reason that LBMs posing a high risk of H5 infection is a combination of virus accumulation at the markets, spread of virus during the trading process and environmental pressures. For effective risk reduction of H5 virus infection at urban LBMs, all three aspects need to be considered. Possible ameliorative measures could include the regulation of poultry trading processes, the relocation of slaughter areas at LMMs to well-managed separate locations and the relocation of wholesale poultry markets outside urban areas.

## Ethical considerations

Data collection for this study was carried out according to the guidelines of the Laboratory of Animal and Fish Health Center (BKHI) Jakarta, Indonesia and was approved by Directorate General of Livestock Services, Indonesia. Market vendors were informed that their participation was strictly voluntarily and verbal consent for the interviews was obtained. The survey responses used in this study were anonymized by the authors. Since all activities were carried out during routine market surveillance no live animals were sampled and no additional animal or human ethics approval was required.

## Supporting information

S1 Table Univariate results for risk factors associated with H5 virus prevalence at live bird markets in the Greater Jakarta Region, between March 2009 and July 2014. (DOCX)

## References

[1] Ding H, Chen Y, Yu Z, Horby PW, Wang F, Hu J, et al A family cluster of three confirmed cases infected with avian influenza A (H7N9) virus in Zhejiang Province of China. BMC Infectious Diseses. 2014;14:698 Epub 2015/01/01. 10.1186/s12879-014-0698-6 25551435 PMC4304124

[2] Gilbert M, Golding N, Zhou H, Wint GR, Robinson TP, Tatem AJ, et al Predicting the risk of avian influenza A H7N9 infection in live-poultry markets across Asia. Nature Communication. 2014;5:4116 Epub 2014/06/18. 10.1038/ncomms5116 24937647 PMC4061699

[3] Waziri NE, Nguku P, Olayinka A, Ajayi I, Kabir J, Okolocha E, et al Evaluating a surveillance system: live-bird market surveillance for highly pathogenic avian influenza, a case study. Pan African Medical Journal. 2014;18 Suppl 1:11 Epub 2014/10/21. 10.11694/pamj.supp.2014.18.1.4188 25328630 PMC4199346

[4] Xu Y, Cao H, Liu H, Sun H, Martin B, Zhao Y, et al Identification of the source of A (H10N8) virus causing human infection. Journal of Molecular Epidemiology and Evolutionary Genetics in Infectious Diseases. 2015;30:159–63. Epub 2015/01/01. 10.1016/j.meegid.2014.12.026 25550151 PMC4838479

[5] Zhou X, Li Y, Wang Y, Edwards J, Guo F, Clements AC, et al The role of live poultry movement and live bird market biosecurity in the epidemiology of influenza A (H7N9): A cross-sectional observational study in four eastern China provinces. The Journal of infection. 2015;71(4):470–9. Epub 2015/07/08. 10.1016/j.jinf.2015.06.012 . 26149187

[6] Wahyono ND, Utami MMD. A Review of the Poultry Meat Production Industry for Food Safety in Indonesia. Journal of Physics Conference Series. 2018;(953):012125 10.1088/1742-6596/953/1/012125

[7] Nathan Associates Inc. Indonesia’s Poultry Value Chain—Costs, Margins, Prices, and Other Issues. United States Agency for International Development. 2013.

[8] Fournie G, Guitian FJ, Mangtani P, Ghani AC. Impact of the implementation of rest days in live bird markets on the dynamics of H5N1 highly pathogenic avian influenza. Journal of the Royal Society Interface. 2011;8(61):1079–89. Epub 2010/12/07. 10.1098/rsif.2010.0510 21131332 PMC3119874

[9] Pepin KM, Wang J, Webb CT, Hoeting JA, Poss M, Hudson PJ, et al Anticipating the prevalence of avian influenza subtypes H9 and H5 in live-bird markets. PloS one. 2013;8(2):e56157 Epub 2013/02/15. 10.1371/journal.pone.0056157 23409145 PMC3567063

[10] Turner JC, Feeroz MM, Hasan MK, Akhtar S, Walker D, Seiler P, et al Insight into live bird markets of Bangladesh: an overview of the dynamics of transmission of H5N1 and H9N2 avian influenza viruses. Emerging Microbes and Infections. 2017;6(3):e12 Epub 2017/08/10. 10.1038/emi.2016.142 28270655 PMC5378921

[11] Yuan J, Lau EH, Li K, Leung YH, Yang Z, Xie C, et al Effect of Live Poultry Market Closure on Avian Influenza A(H7N9) Virus Activity in Guangzhou, China, 2014. Emerging infectious diseases. 2015;21(10):1784–93. Epub 2015/09/25. 10.3201/eid2110.150623 26402310 PMC4593444

[12] Fournie G, Guitian J, Desvaux S, Mangtani P, Ly S, Cong VC, et al Identifying live bird markets with the potential to act as reservoirs of avian influenza A (H5N1) virus: a survey in northern Viet Nam and Cambodia. PloS one. 2012;7(6):e37986 Epub 2012/06/08. 10.1371/journal.pone.0037986 22675502 PMC3366999

[13] Indriani R, Samaan G, Gultom A, Loth L, Indryani S, Adjid R, et al Environmental sampling for avian influenza virus A (H5N1) in live-bird markets, Indonesia. Emerging infectious diseases. 2010;16(12):1889–95. Epub 2010/12/03. 10.3201/eid1612.100402 21122218 PMC3294595

[14] Biswas PK, Giasuddin M, Nath BK, Islam MZ, Debnath NC, Yamage M. Biosecurity and Circulation of Influenza A (H5N1) Virus in Live-Bird Markets in Bangladesh, 2012. Transboundary and emerging diseases. 2015 Epub 2015/12/15. 10.1111/tbed.12454 . 26663031

[15] Pinsent A, Pepin KM, Zhu HC, Guan Y, White MT, Riley S. The persistence of multiple strains of avian influenza in live bird markets. P Roy Soc B-Biol Sci. 2017;284(1868). ARTN 2017071510.1098/rspb.2017.0715. WOS:000417194500002. 10.1098/rspb.2017.0715 PMC5740266 29212718

[16] Zhou L, Ren RQ, Ou JM, Kang M, Wang XX, Havers F, et al Risk Factors for Influenza A(H7N9) Disease in China, a Matched Case Control Study, October 2014 to April 2015. Open Forum Infect Di. 2016;3(3). 10.1093/ofid/ofw182 WOS:000388571700066. 27704029 PMC5047420

[17] Heine HG, Trinidad L, Selleck P, Lowther S. Rapid detection of highly pathogenic avian influenza H5N1 virus by TaqMan reverse transcriptase-polymerase chain reaction. Avian Diseases. 2007;51(1 Suppl):370–2. 10.1637/7587-040206R.1 . 17494586

[18] Clopper CJ, Pearson ES. The use of confidence or fiducial limits illustrated in the case of the binomial. Biometrika. 1934;26:404–13.

[19] Önder H, Olfaz M, Soydan E. Comparison of Working Correlation Matrices in Generalized Estimating Equations for Animal Data2010. 197–201 p.

[20] Li L, Shen C, Li X, Robins JM. On weighting approaches for missing data. Statistical methods in medical research. 2013;22(1):14–30. Epub 2011/06/28. 10.1177/0962280211403597 21705435 PMC3998729

[21] Liu T, Bai Z, Zhang B. Weighted estimating equation: modified GEE in longitudinal data analysis. Frontiers of Mathematics in China. 2014;9(2):329–53. 10.1007/s11464-014-0359-5

[22] Leo L, Marius G, Wu JM, Christina C, Muhammad H, Xiao XM. Identifying risk factors of highly pathogenic avian influenza (H5N1 subtype) in Indonesia. Preventive veterinary medicine. 2011;102(1):50–8. 10.1016/j.prevetmed.2011.06.006 WOS:000294943700006. 21813198 PMC4868053

[23] Yupiana Y, de Vlas SJ, Adnan NM, Richardus JH. Risk factors of poultry outbreaks and human cases of H5N1 avian influenza virus infection in West Java Province, Indonesia. International Journal of Infectious Diseases. 2010;14(9):E800–E5. 10.1016/j.ijid.2010.03.014 WOS:000282101100013. 20637674

[24] Horm VS, Gutiérrez RA, Nicholls JM, Buchy P. Highly Pathogenic Influenza A(H5N1) Virus Survival in Complex Artificial Aquatic Biotopes. PloS one. 2012;7(4). 10.1371/journal.pone.0034160 . 22514622 PMC3325971

[25] Aviat F, Gerhards C, Rodriguez-Jerez JJ, Michel V, Le Bayon I, Ismail R, et al Microbial Safety of Wood in Contact with Food: A Review. Compr Rev Food Sci F. 2016;15(3):491–505. 10.1111/1541-4337.12199 WOS:000373680000003. 33401823

[26] Mariani C, Oulahal N, Chamba JF, Dubois-Brissonnet F, Notz E, Briandet R. Inhibition of Listeria monocytogenes by resident biofilms present on wooden shelves used for cheese ripening. Food Control. 2011;22(8):1357–62. 10.1016/j.foodcont.2011.02.012 WOS:000290505700033.

[27] Henning J, Wibawa H, Morton J, Usman TB, Junaidi A, Meers J. Scavenging Ducks and Transmission of Highly Pathogenic Avian Influenza, Java, Indonesia. Emerging infectious diseases. 2010;16(8):1244–50. 10.3201/eid1608.091540 WOS:000280452600008. 20678318 PMC3298304

[28] Wibawa H, Henning J, Wong F, Selleck P, Junaidi A, Bingham J, et al A molecular and antigenic survey of H5N1 highly pathogenic avian influenza virus isolates from smallholder duck farms in Central Java, Indonesia during 2007–2008. Virol J. 2011;8. Artn 425 10.1186/1743-422x-8-425. WOS:000295366400001. 10.1186/1743-422X-8-425 PMC3179459 21896207

[29] Trock SC, Gaeta M, Gonzalez A, Pederson JC, Senne DA. Evaluation of routine depopulation, cleaning, and disinfection procedures in the live bird markets, New York. Avian Dis. 2008;52(1):160–2. Epub 2008/05/08. 10.1637/7980-040607-Reg . 18459316

