# Correction: Risk factors for H5 avian influenza virus prevalence on urban live bird markets in Jakarta, Indonesia—Evaluation of long-term environmental surveillance data

**DOI:** 10.1371/journal.pone.0221611

**Published:** 2019-08-20

**Authors:** The PLOS ONE Staff

In Table 1, the headings Mean (SD) H5 negative and Mean (SD) H5 positive do not appear in the correct columns. The publisher apologizes for the error. Please see the correct Table 1 here.

**Table 1:** Final multivariable model results for risk factors associated with HPAI H5 virus prevalence at live bird markets in the Greater Jakarta Region, Indonesia, between March 2009 and July 2014.

RISK FACTORS	Level	N Observations (percent)		Univariate analysis			Multivariable analysis		
		H5 negative	H5 positive	OR	P-value	Wald test P-value	OR	P-value	Wald test P-value
**MARKET CHARACTERISTICS**		** **							
Market layout^1^	A	934 (64.3%)	518 (35.7%)	1		<0.001	1		<0.001
	B	537 (58.3%)	384 (35.7%)	1.3 (0.8, 2.3)	0.322		1.1 (0.7, 1.6)	0.786	
	C	169 (56.1%)	132 (43.9%)	1.3 (0.8, 2.0)	0.268		1.5 (1.0, 2.2)	0.060	
	D	33 (34.4%)	63 (65.6%)	3.3 (2.3, 4.9)	<0.001		1.4 (0.8, 2.3)	0.229	
	E	58 (57.4%)	43 (42.6%)	1.3 (0.7, 2.4)	0.358		1.1 (0.5, 2.2)	0.829	
	F	120 (79.5%)	31 (20.5%)	0.3 (0.1, 0.9)	0.03		0.4 (0.2, 1.1)	0.071	
	SALE only 1	159 (56.0%)	125 (44.0%)	1.4 (0.7, 2.8)	0.377		1.3 (0.7, 2.1)	0.417	
	SALE only 2	254 (93.0%)	19 (7.0%)	0.1 (0.0, 0.3)	<0.001		0.2 (0.1, 0.5)	0.002	
Most dominant poultry species on the market	Broilers	1,592 (66.4%)	807 (33.6%)	1		<0.001	1		<0.001
	Layers	92 (66.2%)	47 (33.8%)	1.0 (0.7, 1.5)	0.849		0.7 (0.5, 1.0)	0.054	
	Kampung Chickens	435 (56.3%)	337 (43.7%)	1.5 (0.8, 2.9)	0.243		1.3 (0.8, 2.2)	0.355	
	Ducks	120 (54.5%)	100 (45.5%)	1.6 (0.8, 3.0)	0.149		0.9 (0.5, 1.9)	0.868	
	Parent stock	25 (51.0%)	24 (49.0%)	2.0 (1.5, 2.7)	<0.001		5.7 (3.6, 9.2)	<0.001	
**POULTRY MANAGEMENT ON MARKET**									
Display tables made from wood	No	1,032 (58.3%)	739 (41.7%)	1					
	Yes	1,232 (68.1%)	576 (31.9%)	0.6 (9.3, 0.9)	0.018		0.7 (0.5, 1.0)	0.047	
**SAMPLING CHARACTERISTICS**									
Samples obtained from at least one duck	No	2,083 (65.8%)	1,083 (34.2%)	1					
	Yes	181 (43.8%)	232 (56.2%)	1.5 (1.1, 2.1)	0.019		1.6 (1.1, 2.3)	0.009	
**ENVIRONMENTAL FACTORS**		**Mean (SD)**							
		**H5 negative**	**H5 positive**						
Human population density in the district (N people/square kilometre)		9,018.3 (5,023.0)	11,958.9 (4,379.6)	2.2 (1.8, 2.7)^2^	<0.001		1.6 (1.3, 1.9)^2^	<0.001	
Average distance between market and origin of poultry sold at the market (kilometre)		16.8 (35.7)	35.9 (47.8)	1.6 (1.2, 2.0)^2^	<0.001		1.3 (1.1, 1.6)^2^	0.011	
Total rainfall per month (mm)		94.2 (87.1)	113.3 (94.4)	1.2 (1.2, 1.3)^2^	<0.001		1.3 (1.2, 1.4)^2^	<0.001	

^1^ Live Bird Market layouts: A = Slaughter, sale of live birds and carcass sales in same area, B = Slaughter and live bird sales conducted outside and carcass sales inside, C = Slaughter, sale of live birds and carcass sales in same areas, but separated by individual partitions, D = Slaughter and live bird sales in same areas inside, and separated from carcass sales by screens, E = Slaughter, live bird and carcass sales inside in separate areas, but no protective screens, F = Live birds sale outside and slaughter and carcass sales inside and in separate areas, Sale 1 only = No slaughtering at the market, but slaughter facility in vicinity of the market, Sale 2 only = No slaughtering at the market, and slaughter facility far away from the market

^2^ Odds ratios and confidence intervals are displayed for standardized values
