# Supplementary material for: Risk factors for H5 avian influenza virus prevalence on urban live bird markets in Jakarta, Indonesia—Evaluation of long-term environmental surveillance data
Source: PLoS One. 2019 May 24;14(5):e0216984. doi: 10.1371/journal.pone.0216984 (PMC6534305; doi:10.1371/journal.pone.0216984)
Supplement: S1 Table — (DOCX) [file pone.0216984.s001.docx]

**S1 Table: Univariate results for risk factors associated with H5 virus prevalence at live bird markets in the Greater Jakarta Region, between March 2009 and July 2014.**

|  |  | **N Observations (percent)** | | | **Univariate analysis results** | | | | |
| --- | --- | --- | --- | --- | --- | --- | --- | --- | --- |
| **Risk factor** | **Level** | **H5 negative** | **H5 positive** | | **OR** | **OR 95% CI low** | **OR 95% CI high** | **P-value** | **Wald test P-value** |
| **MARKET CHARACTERISTICS** | | | | |  |  |  |  |  |
| Market location | | | | |  |  |  |  |  |
|  | Bogor | 330 (93.8%) | | 22 (6.3%) | 1.0 |  |  |  | <0.001 |
|  | Bekasi | 135 (67.5%) | | 65 (32.5%) | 8.2 | 2.4 | 27.8 | 0.001 |  |
|  | Jakarta Barat | 34 (30.9%) | | 76 (69.1%) | 47.4 | 16.0 | 140.6 | <0.001 |  |
|  | Jakarta Pusat | 79 (41.1%) | | 113 (58.9%) | 28.7 | 9.9 | 83.1 | <0.001 |  |
|  | Jakarta Selatan | 155 (49.7%) | | 157 (50.3%) | 18.0 | 5.8 | 56.0 | <0.001 |  |
|  | Jakarta Timur | 52 (21.4%) | | 191 (78.6%) | 72.3 | 23.2 | 225.5 | <0.001 |  |
|  | Jakarta Uttara | 152 (65.5%) | | 80 (34.5%) | 10.5 | 3.8 | 29.1 | <0.001 |  |
|  | Kota Bekasi | 205 (54.1%) | | 174 (45.9%) | 16.4 | 6.0 | 45.1 | <0.001 |  |
|  | Kota Bogor | 71 (68.9%) | | 32 (31.1%) | 11.0 | 2.0 | 60.6 | 0.006 |  |
|  | Kota Depok | 84 (55.3%) | | 68 (44.7%) | 16.6 | 5.6 | 49.8 | <0.001 |  |
|  | Kota Tangerang | 351 (73.3%) | | 128 (26.7%) | 7.3 | 2.6 | 20.9 | <0.001 |  |
|  | Tangerang | 334 (75.4%) | | 109 (24.6%) | 6.6 | 2.4 | 18.3 | <0.001 |  |
|  | Tangerang Selatan | 282 (73.8%) | | 100 (26.2%) | 7.3 | 2.5 | 21.4 | <0.001 |  |
| Market layout | | | | |  |  |  |  |  |
|  | A | 934 (64.3%) | | 518 (35.7%) | 1.0 |  |  |  | <0.001 |
|  | B | 537 (58.3%) | | 384 (35.7%) | 1.3 | 0.8 | 2.3 | 0.322 |  |
|  | C | 169 (56.1%) | | 132 (43.9%) | 1.3 | 0.8 | 2.0 | 0.268 |  |
|  | D | 33 (34.4%) | | 63 (65.6%) | 3.3 | 2.3 | 4.9 | <0.001 |  |
|  | E | 58 (57.4%) | | 43 (42.6%) | 1.3 | 0.7 | 2.4 | 0.358 |  |
|  | F | 120 (79.5%) | | 31 (20.5%) | 0.3 | 0.1 | 0.9 | 0.030 |  |
|  | SALE 1 | 159 (56.0%) | | 125 (44.0%) | 1.4 | 0.7 | 2.8 | 0.377 |  |
|  | SALE 2 | 254 (93.0%) | | 19 (7.0%) | 0.1 | 0.0 | 0.3 | 0.000 |  |
| Most dominant poultry species on the market | | | | |  |  |  |  |  |
|  | Broilers | 1592 (66.4%) | | 807 (33.6%) | 1.0 |  |  |  | <0.001 |
|  | Layers | 92 (66.2%) | | 47 (33.8%) | 1.0 | 0.7 | 1.5 | 0.849 |  |
|  | Kampung Chickens | 435 (56.3%) | | 337 (43.7%) | 1.5 | 0.8 | 2.9 | 0.243 |  |
|  | Ducks | 120 (54.5%) | | 100 (45.5%) | 1.6 | 0.8 | 3.0 | 0.149 |  |
|  | Parent stock | 25 (51%) | | 24 (49.0%) | 2.0 | 1.5 | 2.7 | <0.001 |  |
| Trading volume | | | | |  |  |  |  |  |
|  | <1000 | 769 (74.1%) | | 269 (25.9%) | 1.0 |  |  |  | 0.003 |
|  | >1000-5000 | 1,058 (61.0%) | | 676 (39.0%) | 2.6 | 1.5 | 4.7 | 0.001 |  |
|  | >5000 | 437 (54.2%) | | 370 (45.8%) | 2.8 | 1.5 | 5.4 | 0.002 |  |
| **POULTRY MANAGEMENT ON MARKET** | | | | |  |  |  |  |  |
| Poultry kept in wooden cages | | | | |  |  |  |  |  |
|  | No | 1804 (67.8%) | | 858 (32.2%) | 1.0 |  |  |  |  |
|  | Yes | 460 (50.2%) | | 457 (49.8%) | 2.0 | 1.1 | 3.6 | 0.020 |  |
| Poultry kept in plastic cages | | | | |  |  |  |  |  |
|  | No | 1376 (59.5%) | | 935 (40.5%) | 1.0 |  |  |  |  |
|  | Yes | 888 (70.0%) | | 380 (30%) | 0.6 | 1.0 | 0.1 | 0.078 |  |
| Poultry kept in bamboo cages | | | | |  |  |  |  |  |
|  | No | 1,051 (62.1%) | | 642 (37.9%) | 1.0 |  |  |  |  |
|  | Yes | 1,213 (64.3%) | | 673 (35.7%) | 1.1 | 0.7 | 1.8 | 0.604 |  |
| Cages placed vertically without separation between cages | | | | |  |  |  |  |  |
|  | No | 1,644 (64.8%) | | 894 (35.2%) | 1.0 |  |  |  |  |
|  | Yes | 620 (59.6%) | | 421 (40.4%) | 1.8 | 1.1 | 2.8 | 0.014 |  |
| Cages placed vertically with separation between cages | | | | |  |  |  |  |  |
|  | No | 1,919 (65.7%) | | 1,003 (34.3%) | 1.0 |  |  |  |  |
|  | Yes | 345 (52.5%) | | 312 (47.5%) | 1.7 | 0.9 | 3.2 | 0.077 |  |
| Cages placed next to each other on the floor | | | | |  |  |  |  |  |
|  | No | 1,601 (59.9%) | | 1,074 (40.1%) | 1.0 |  |  |  |  |
|  | Yes | 663 (73.3%) | | 241 (26.7%) | 0.4 | 0.3 | 0.8 | 0.005 |  |
| Cages placed next to each other on the floor, but with separation between the floor and the cage | | | | |  |  |  |  |  |
|  | No | 1,850 (64.1%) | | 1,034 (35.9%) | 1.0 |  |  |  |  |
|  | Yes | 414 (59.6%) | | 281 (40.4%) | 1.2 | 0.7 | 2.0 | 0.587 |  |
| Display tables made from stainless steel or ceramic | | | | |  |  |  |  |  |
|  | No | 453 (54.8%) | | 373 (45.2%) | 1.0 |  |  |  |  |
|  | Yes | 1,811 (65.8%) | | 942 (34.2%) | 0.8 | 0.4 | 1.4 | 0.348 |  |
| Display tables made from wood | | | | |  |  |  |  |  |
|  | No | 1032 (58.3%) | | 739 (41.7%) | 1.0 |  |  |  |  |
|  | Yes | 1232 (68.1%) | | 576 (31.9%) | 0.6 | 0.3 | 0.9 | 0.018 |  |
| Slaughter at the market | | | | |  |  |  |  |  |
|  | No | 413 (74.1%) | | 144 (25.9%) | 1.0 |  |  |  |  |
|  | Yes | 1851 (61.3%) | | 1171 (38.7%) | 2.4 | 1.0 | 5.5 | 0.041 |  |
| Slaughter on the floor | | | | |  |  |  |  |  |
|  | No | 1,271 (63.5%) | | 731 (36.5%) | 1.0 |  |  |  |  |
|  | Yes | 993 (63.0%) | | 584 (37.0%) | 1.1 | 0.7 | 1.7 | 0.739 |  |
| Slaughter on wooden tables | | | | |  |  |  |  |  |
|  | No | 1,400 (61.7%) | | 869 (38.3%) | 1.0 |  |  |  |  |
|  | Yes | 864 (66.0%) | | 446 (34.0%) | 1.0 | 0.6 | 1.6 | 0.919 |  |
| Slaughter and carcass processing (e.g. plucking, disembowelling) in same area by same workers | | | | |  |  |  |  |  |
|  | No | 715 (63.2%) | | 416 (36.8%) | 1.0 |  |  |  |  |
|  | Yes | 1,549 (63.3%) | | 899 (36.7%) | 1.2 | 0.7 | 2.2 | 0.492 |  |
| Slaughter and carcass processing (e.g. plucking, disembowelling) in different area by different workers | | | | |  |  |  |  |  |
|  | No | 2,083 (63.5%) | | 1,195 (36.5%) | 1.0 |  |  |  |  |
|  | Yes | 181 (60.1%) | | 120 (39.9%) | 1.3 | 0.6 | 2.7 | 0.624 |  |
| Birds staying overnight at the market | | | | |  |  |  |  |  |
|  | No | 669 (70.6%) | | 279 (29.4%) | 1.0 |  |  |  |  |
|  | Yes | 1595 (60.6%) | | 1036 (39.4%) | 2.1 | 1.2 | 3.5 | 0.009 |  |
| **SAMPLING CHARACTERISTICS** | | | | |  |  |  |  |  |
| Samples obtained from broiler only | | | | |  |  |  |  |  |
|  | No | 697 (48.7%) | | 735 (51.3%) | 1.0 |  |  |  |  |
|  | Yes | 1567 (73.0%) | | 580 (27.0%) | 0.5 | 0.4 | 0.7 | <0.001 |  |
| Samples obtained from at least one Kampung chicken | | | | |  |  |  |  |  |
|  | No | 1,702 (70.6%) | | 709 (29.4%) | 1.0 |  |  |  |  |
|  | Yes | 562 (48.1%) | | 606 (51.9%) | 1.8 | 1.4 | 2.4 | <0.001 |  |
| Samples obtained from at least one duck | | | | |  |  |  |  |  |
|  | No | 2,083 (65.8%) | | 1,083 (34.2%) | 1.0 |  |  |  |  |
|  | Yes | 181 (43.8%) | | 232 (56.2%) | 1.5 | 1.1 | 2.1 | 0.019 |  |
| Samples from slaughter area only | | | |  |  |  |  |  |  |
|  | No | 1,956 (66.1%) | | 1,002 (39.9%) | 1.0 |  |  |  |  |
|  | Yes | 308 (49.6%) | | 313 (50.4%) | 2.7 | 1.6 | 4.5 | <0.001 |  |
| Samples from display area only | | | |  |  |  |  |  |  |
|  | No | 2,004 (61.5%) | | 1,254 (38.5%) | 1.0 |  |  |  |  |
|  | Yes | 260 (81.0%) | | 61 (19.0%) | 0.2 | 0.1 | 0.7 | 0.010 |  |
| **ENVIRONMENTAL FACTORS** | | | | |  |  |  |  |  |
|  |  | **Mean (SD)** | | **Mean (SD)** | **OR** | **OR 95% CI low** | **OR 95% CI high** | **P-value** |  |
|  |  | **H5 negative** | | **H5 positive** |  |  |  |  |  |
| Density of poultry retail markets in the district (number/square kilometre) | | 0.110 (0.106) | | 0.200 (0.172) | 1.3 | 0.9 | 1.9 | 0.130 |  |
| Density of poultry wholesale markets in the district (number/square kilometre) | | 0.120 (0.213) | | 0.149 (0.197) | 2.1 | 1.8 | 2.6 | <0.001 |  |
| Average distance between market and origin of poultry sold at the market (kilometre) | | 16.8 (35.7) | | 35.9 (47.8) | 1.6 | 1.2 | 2.0 | <0.001 |  |
| Human population density (number of people/square kilometre) | | 9,018.3 (5023.0) | | 11,958.9 (4379.6) | 2.2 | 1.8 | 2.7 | <0.001 |  |
| Total rainfall per month (mm) | | 94.2 (87.1) | | 113.3 (94.4) | 1.2 | 1.2 | 1.3 | <0.001 |  |
